# A prostate-specific antigen doubling time of <6 months is prognostic for metastasis and prostate cancer-specific death for patients receiving salvage radiation therapy post radical prostatectomy

**DOI:** 10.1186/1748-717X-8-170

**Published:** 2013-07-08

**Authors:** William C Jackson, Skyler B Johnson, Darren Li, Corey Foster, Benjamin Foster, Yeohan Song, Matthew Schipper, Mark Shilkrut, Howard M Sandler, Todd M Morgan, Ganesh S Palapattu, Daniel A Hamstra, Felix Y Feng

**Affiliations:** 1 Department of Radiation Oncology, University of Michigan, 1500 E. Medical Center Dr., Ann Arbor, MI 48105, USA; 2 Department of Radiation Oncology, Cedars-Sinai, 8700 Beverly Blvd., Los Angeles, CA 90048, USA; 3 Department of Urology, University of Michigan, 1500 E. Medical Center Dr., Ann Arbor, MI 48105, USA

**Keywords:** Prostate-specific antigen doubling time, Salvage radiation therapy, Prostate cancer

## Abstract

**Background:**

The ideal prostate-specific antigen (PSA) doubling time (PSADT) threshold for identifying patients at high-risk for poor clinical outcome following salvage radiation therapy (SRT) has not been well established. We sought to assess what PSADT threshold is most clinically prognostic in this setting.

**Methods:**

575 patients who received SRT at a single institution for biochemical recurrence after radical prostatectomy were retrospectively reviewed. We assessed the impact of pre-SRT PSADT on biochemical failure (BF), distant metastasis (DM), prostate cancer-specific mortality (PCSM), and overall mortality (OM). Kaplan-Meier methods, hazard ratio (HR) assessment, and Cox Proportional Hazard models were used to assess the discriminatory ability of various PSADT thresholds.

**Results:**

Sufficient data to calculate PSADTs were available for 277 patients. PSADT was prognostic for BF, DM, PCSM, and OM on univariate analysis regardless of threshold. HR assessment identified 6 months as a strong threshold. No statistically significant difference was observed in BF, DM, PCSM, or OM between patients with PSADT <3 (n=40) and 3–6 months (n=61) or between 6–10 (n=62) and >10 months (n=114). However significant differences were seen in BF (HR:2.2, [95%CI: 1.4-3.5], p<0.01) and DM (HR:2.2, [95%CI: 1.2-4.3], p=0.02) between a PSADT of 3–6 and 6–10 months. On multivariate analysis a PSADT <6 months predicted BF (HR:2.0, [95%CI: 1.4-2.9], p=0.0001), DM (HR:2.0, [95%CI: 1.2-3.4], p=0.01), and PCSM (HR:2.6, [95%CI: 1.1-5.9], p=0.02).

**Conclusions:**

A pre-SRT PSADT <6 months was a strong predictor of outcomes in our data set, including PCSM. The most common nomogram for SRT uses a 10-month PSADT threshold for assigning points used to assess BF following SRT. If validated, our findings suggest that a PSADT threshold of <6 months should be considered for stratification of patients in future clinical trials in this setting.

## Background

Each year >150,000 patients in the United States will undergo radical prostatectomy (RP) for prostate cancer [1]. Up to 30% of these patients will eventually experience biochemical failure (BF) as manifested by a rising prostate-specific antigen (PSA) [2]. A rising PSA after RP can be indicative of local recurrence, distant disease, or both. Many patients who experience BF post-RP will go on to receive salvage external beam radiation therapy (SRT). Unfortunately, a suboptimal number of patients undergoing SRT obtain long-term recurrence free responses [3]. It is likely that many of the patients who experience biochemical failure (BF) after SRT represent a subset of patients who harbor micrometastatic disease at the time of SRT. Improved prognostic factors are needed to better delineate the patients who have locally confined disease and are thus most likely to respond favorably to SRT.

A short PSA doubling time (PSADT) is known to predict both the development of metastasis (DM), prostate cancer-specific mortality (PCSM), and increased overall mortality (OM) in patients experiencing BF after RP [4-10]. Similarly, a short PSADT after RP predicts worse clinical outcomes following SRT, including an increased likelihood of BF and development of distant metastasis [3,11-18]. To our knowledge, a direct association between a short pre-SRT PSADT and increased PCSM following SRT has yet to be reported; however, others have noted an association between a short pre-SRT PSADT and an improved overall benefit of SRT [19,20]. Furthermore, the most clinically useful PSADT threshold for defining high-risk patients has not been well established. Frequently proposed PSADT cut-points include 3, 6, 10, and 12 months [3,12-14,19]. A well-known predictive tool for response to SRT, the Stephenson nomogram and decision tree, utilizes a PSADT threshold of 10 months [3].

We sought to determine the most clinically prognostic pre-SRT PSADT cut-point for patients receiving SRT following RP.

## Methods

### Patient selection

Through an institutional review board approved analysis, 575 patients who received SRT at a single institution, with or without androgen deprivation therapy (ADT), for BF following RP were identified and retrospectively reviewed. All patients received SRT between 1986 and 2010. SRT was defined as radiation therapy (RT) given for a persistently elevated PSA ≥0.2 ng/mL post-RP and any RT given for BF post-RP. Of the 575 SRT patients, 277 had sufficient PSA data available following BF to calculate PSADTs and comprise the cohort for this analysis. PSADTs were calculated as previously defined [4]. All available PSA values from BF post-RP and the start of SRT were used to calculate PSADT. Ultrasensitive PSA values were not used to calculate PSADT. If a patient was started on neoadjuvant ADT prior to SRT, PSADT was calculated using only PSA values available before the start of ADT.

### Treatment

SRT was delivered using either three-dimensional conformal radiation therapy or intensity-modulated radiation therapy. Prescribed SRT doses ranged from 64.8-68.4 Gy, typically to the prostate bed only, with whole-pelvic radiation therapy (WPRT) used in only 10% of patients. The use of ADT was per physician discretion and was utilized in 15% of patients. Standard patient follow-up consisted of appointments 3–4 times per year for the first 2 years, then twice a year through year 5, and annually thereafter.

### Endpoints

The outcomes of interest for this study include biochemical failure (BF), development of distant metastasis (DM), prostate cancer-specific mortality (PCSM), and overall mortality (OM). BF was defined as a serum PSA 0.2 ng/mL greater than the post-SRT PSA nadir, followed by a consecutively higher PSA value [3]. DM was defined as the development of clinical, radiologic, or pathologic evidence of metastasis. PCSM was defined as any death in a patient with metastasis or hormone refractory disease, or any death attributed to prostate cancer. OM was defined as death from any cause.

### Statistical analysis

The Chi-square test was utilized to compare categorical variables. One-way analysis of variance (ANOVA) was used for comparison of continuous variables. Concordance index (c-index) values were calculated to quantify the discriminatory ability of PSADT as a continuous variable for predicting time to the various survival outcomes [21]. Ties in the predictor were not common given the continuous nature of PSADT but were included in the calculation as 0.5 in the numerator. Kaplan-Meier methods, hazard ratio (HR) assessment, and Cox Proportional Hazard models were used to evaluate various PSADT thresholds. All statistical analysis was performed using MedCalc (v12.3.0.0, MedCalc Software, Mariakerke, Belguim).

## Results

### Patient characteristics

Sufficient data to calculate PSADTs was available for 277 patients (48%). Of the patients with available PSADTs, median age was 65 years and median age-adjusted Charlson co-morbidity index (CCMI) was 3 [22]. The median time of follow up post-SRT was 83 months and median RT dose was 68.4 Gy (interquartile range [IQR] 64.8-68.4 Gy). The median PSADT was 7.9 months (IQR 4.3-15.8). Overall, 40 patients had a PSADT <3 months, 61 had a PSADT between 3 and 6 months, 62 had a PSADT between 6 and 10 months, 114 had a PSADT >10 months, 96 had a PSADT >12 months and only 18 patients with a PSADT between 10 and 12 months.

Patient characteristics were stratified by a PSADT cut-off of 6 months as this was the closest proposed threshold to our median PSADT (Table 1). 101 patients had a PSADT <6 months, and 176 had a PSADT >6 months. No difference existed between the two groups with respect to age, CCMI, SRT dose, use of WPRT, use of ADT, ADT duration, pathologic T-stage, seminal vesicle involvement (SVI), extra-capsular extension (ECE), presence of surgical margins (SM), or lymph node invasion (LNI). Patients with PSADT <6 months did have a statistically significant higher pre-SRT PSAs (p<0.001). The difference in median pre-SRT PSA was only 0.2 ng/mL higher for patients with PSADTs <6 months and the groups had overlapping interquartile ranges. Patients with PSADT <6 months were also more likely to have a Gleason score of 8 to 10 than patients with PSADTs >6 months (p=0.02).

**Table 1:** Patient pathologic, pre-treatment, and treatment characteristics

	**PSADT**		
	**PSADT > 6**	**PSADT < 6**	**p-value**
	**(n=176)**	**(n=101)**	
Age Median (IQR)	65.5 (59.4-70.2)	62.8 (58.6-68.6)	p=0.14*
CCMI Median (IQR)	3.0 (3.0-4.0)	3.0 (3.0-4.0)	p=0.2*
Range	1.0-8.0	1.0-7.0	
Pre-SRT PSA Median (IQR)	0.6 (0.4-1.1)	0.8 (0.5-1.75)	p<0.001*
Range	0.1-6.3	0.2-17.4	
SRT dose Median (IQR)	68.4 (64.8-68.4)	68.4 (64.8-68.4)	p=0.05*
WPRT	11.4%	6.9%	p=0.3**
ADT during SRT	12.5%	18.8%	p=0.2**
ADT duration	5.9 (4.4-8.5)	6.1 (4.7-15.1)	p=0.6*
Gleason score			
2-6	22.2%	11.9%	p=0.02**
7	61.4%	60.4%	
8-10	16.5%	27.7%	
pTstage			
T1-T2a	13.0%	6.3%	p=0.15**
T2b-T2c	32.2%	27.5%	
T3-T4	54.8%	66.3%	
SVI	9.8%	16.8%	p=0.13**
ECE	45.4%	52.5%	p=0.3**
SM	43.2%	44.0%	p=0.9**
LNI	2.1%	1.2%	p=0.9**

Abbreviations: *PSADT* Prostate-specific antigen doubling time, *PSA* Prostate-specific antigen, *IQR* Interquartile range, *CCMI* Charlson comorbidity index, *SRT* Salvage radiation therapy, *WPRT* Whole-pelvic radiation therapy, *ADT* Androgen deprivation therapy, *pTstage* Pathologic T stage, *SVI* Seminal vesicle invasion, *ECE* Extra-capsular extension, *SM* Surgical margin, *LNI* Lymph node involvement, *Analysis of variance, ** Chi-square.

### PSADT threshold determination

**Figure 1:**
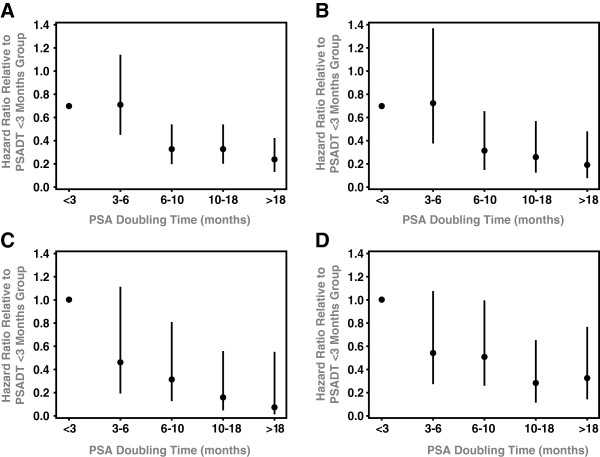
**Analysis of Hazard Ratios.** Hazard ratio comparisons with 95% confidence intervals for various PSADT thresholds for **A)** Biochemical Failure, **B)** Metastasis, **C)** Prostate cancer-specific mortality, **D)** Overall mortality.

Using c-indices, PSADT was evaluated as a continuous variable to establish its discriminatory ability. PSADT did have prognostic significance for each end-point including: BF (0.623), DM (0.670), PCSM (0.721), and OM (0.636). Having established the prognostic association, we sought to optimize a PSADT threshold. Hazard ratios (HR) with 95% confidence intervals were calculated for a range of PSADT thresholds (<3, 3–6, 6–10, 10–18, and >18 months) relative to patients with a PSADT <3 months for BF, DM, PCMS, and OM (Figure 1). From these figures, it is apparent that BF and DM outcomes in the 3–6 month group were very similar to the 0–3 month group, and outcomes in the 6–10 month group were very similar to those of the 10–18 and >18 month groups. This motivated use of a 6 month threshold as opposed to 3 or 10 months. Analysis of these figures also demonstrated that 6 months is the ideal threshold for determining patients at highest risk for BF (HR:2.3, [95%CI: 1.6-3.3], p<0.01) and DM (HR:2.8, [95%CI: 1.7-4.6], p<0.01), as 6 months discriminates between those at high-risk for BF and DM (PSADT <6 months) and low-risk for BF and DM (>6 months). For PCSM, there appears to be a more continuous decline in risk with increasing PSADT, with a particularly notable difference in the 0–3 and the 3–6 month groups. Nevertheless, 6 months still appears to be a suitable threshold for this outcome as well, as the decline in risk for those with a PSADT >6 months is significantly less steep than the increase in risk for PCSM for those with a PSADT <6 months. An ideal cut-point for determining patients at highest risk for OM was less obvious using this method.

### Univariate analysis

**Figure 2:**
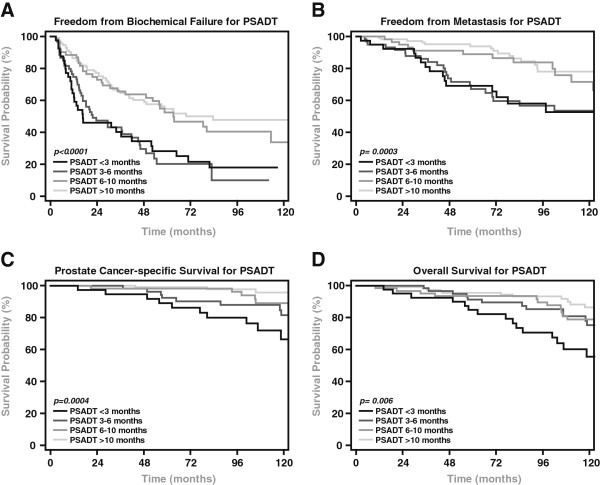
**Kaplan-Meier Analysis of PSA Doubling Time.** Freedom from biochemical failure **(A)**, freedom from metastasis **(B)**, cause-specific survival **(C)**, and overall survival **(D)** for patients stratified by PSADT.

By log-rank analysis, a short PSADT significantly predicted BF, DM, PCSM, and OM regardless of cut-off point (3, 6, or 10 months, all p <0.05). Other significant predictors of outcome on univariate analysis included pre-SRT PSA, Gleason score, and SVI (Table 2). Patients with PSADTs <3, 3–6, 6–10, and >10, were compared (Figure 2). By log-rank test, there was no statistical difference in BF, DM, PCSM, or OM between patients with PSADT <3 (n=40) and 3–6 months (n=61), and similarly, no difference existed between those with a PSADT of 6–10 (n=62) and >10 months (n=114). A difference did exist in BF (HR:2.2, [95%CI: 1.4-3.5], p<0.01) and DM (HR:2.2, [95%CI: 1.2-4.3], p=0.02) for patients with a PSADT between 3–6 as compared to 6–10 months.

**Table 2:** Univariate analysis

	**BF**	**DM**	**PCSM**	**OM**
PSADT < 6 months	HR: 2.3	HR: 2.8	HR: 3.3	HR: 1.80
	[95%CI: 1.6-3.3]	[95%CI: 1.7-4.6]	[95%CI: 1.6-6.9]	[95%CI: 1.1-3.0]
	p<0.0001	p<0.0001	p=0.0007	p=0.01
Pre-SRT PSA	HR: 1.2	HR: 1.2	HR: 1.2	HR: 1.2
	[95%CI: 1.1-1.3]	[95%CI: 1.1-1.3]	[95%CI: 1.1-1.3]	[95%CI: 1.01-1.2]
	p<0.0001	p<0.0001	p<0.0001	p=0.0003
ADT during SRT	HR: 0.8	HR: 0.9	HR: 0.9	HR: 1.1
	[95%CI: 0.5-1.2]	[95%CI: 0.46-1.77]	[95%CI: 0.3-2.4]	[95%CI: 0.6-2.3]
	p=0.3	p=0.8	p=0.8	p=0.7
Gleason score*	HR: 1.5	HR: 1.8	HR: 2.0	HR: 1.2
	[95%CI: 1.2-1.7]	[95%CI: 1.4-2.4]	[95%CI: 1.4-2.9]	[95%CI: 0.9-1.5]
	p<0.0001	p<0.0001	p=0.0002	p=0.18
SVI	HR: 2.2	HR: 2.3	HR: 3.2	HR: 2.2
	[95%CI: 1.3-3.7]	[95%CI: 1.1-4.9]	[95%CI: 1.1-9.9]	[95%CI: 1.0-4.7]
	p=0.0001	p=0.0034	p=0.002	p=0.008
ECE	HR: 1.8	HR: 1.5	HR: 1.7	HR: 1.6
	[95%CI: 1.3-2.5]	[95%CI: 0.9-2.4]	[95%CI: 0.8-3.4]	[95%CI: 0.97-2.6]
	p=0.0004	p=0.12	p=0.16	p=0.07
SM	HR: 0.6	HR: 0.7	HR: 1.2	HR: 0.9
	[95%CI: 0.5-0.9]	[95%CI: 0.5-1.2]	[95%CI: 0.6-2.4]	[95%CI: 0.5-1.4]
	p=0.005	p=0.3	p=0.7	p=0.5
LNI	HR: 1.2	HR: 2.0	HR: 3.6	HR: 1.9
	[95%CI: 0.4-4.3]	[95%CI: 0.3-14.1]	[95%CI: 0.3-47.7]	[95%CI: 0.3-12.9]
	p=0.7	p=0.3	p=0.06	p=0.4

Abbreviations: *BF*; Biochemical failure, *DM*; Distant metastasis, *PCSM*; Prostate cancer-specific survival, *OM*; Overall mortality, *PSADT*; Prostate-specific antigen doubling time, *PSA*; Prostate-specific antigen, *SRT*; Salvage radiation therapy, *SVI*; Seminal vesicle invasion, *ECE*; Extra-capsular extension, *SM*; Surgical margin, *LNI*; Lymph node involvement, *HR;* Hazard ratio, *CI;* Confidence interval, *Cox-proportional hazards.

Lastly, patients with a PSADT <6 months were compared on univariate analysis to those with a PSADT >6 months. Patients with a PSADT <6 months were at a significantly increased risk for BF (HR:2.3, [95%CI: 1.6-3.3], p<0.01), DM (HR:2.8, [95%CI: 1.7-4.6], p<0.01), PCSM (HR:3.3, [95%CI: 1.6-6.9], p<0.01), and had increased OM (HR:1.8, [95%CI: 1.1-3.0], p=0.01) compared to the patients with a PSADT >6 months.

### Multivariate analysis

Multivariate analysis was performed using PSADT <6 months, pre-SRT PSA, Gleason score, SVI, ECE, and SM (Table 3). After adjusting for other clinical variables and controlling for SRT dose, the use of WPRT, and age-adjusted CCMI, a PSADT <6 months was prognostic for BF (HR:2.0, [95%CI: 1.4-2.9], p=0.0001), DM (HR:2.0, [95%CI: 1.2-3.4], p=0.01), and PCSM (HR:2.6, [95%CI: 1.1-5.9], p=0.02).

**Table 3:** Multivariate analysis

	**Biochemical failure**			**Metastasis**			**Prostate cancer-specific mortality**			**Overall mortality**		
**Variable**	**HR**	**95%CI**	**p-value**	**HR**	**95%CI**	**p-value**	**HR**	**95%CI**	**p-value**	**HR**	**95%CI**	**p-value**
PSADT <6 months	2.0	1.4-2.9	0.0001	2.0	1.2-3.4	0.01	2.6	1.1-5.9	0.02	1.4	0.8-2.4	0.3
Pre-SRT PSA	1.1	1.03-1.2	0.01	1.2	1.1-1.3	0.001	1.2	1.1-1.3	0.003	1.2	1.1-1.3	0.001
Gleason score	1.2	1.01-1.5	0.03	1.6	1.2-2.1	0.002	1.6	1.0-2.7	0.04	0.9	0.7-1.3	0.6
SVI	1.4	0.8-2.2	0.2	1.6	0.8-3.3	0.2	2.0	0.7-5.6	0.2	1.7	0.8-3.7	0.2
ECE	1.6	1.1-2.4	0.01	1.1	0.6-2.0	0.7	0.7	0.3-1.9	0.6	1.5	0.8-2.9	0.2
SM	0.5	0.4-0.7	0.0002	0.6	0.3-0.9	0.03	1.0	0.4-2.1	0.9	0.7	0.4-1.2	0.2
SRT dose	1.0	0.9-1.1	0.5	1.0	0.9-1.2	0.5	0.8	0.7-1.0	0.07	0.9	0.8-1.0	0.2
WPRT	0.7	0.4-1.5	0.4	1.6	0.6-3.9	0.3	1.5	0.4-5.7	0.5	1.3	0.5-3.3	0.6
Age-adjusted CCMI	1.0	0.9-1.2	0.7	1.1	0.9-1.3	0.6	1.3	0.9-1.8	0.1	1.3	1.1-1.7	0.01

Abbreviations: *PSADT*; prostate-specific antigen doubling time, *PSA*; prostate-specific antigen, *SRT*; salvage radiation therapy, *SVI*; seminal vesicle invasion, *ECE*; extra-capsular extension, *SM*; surgical margin, *WPRT*; whole pelvis radiation therapy, *CCMI*; charlson co-morbidity index, *HR*; hazard ratio, *CI*; confidence interval.

## Discussion

PSADT after BF following RP is prognostic for clinical outcomes as well as response to SRT [3-18]. Boorjian et al. found that a PSADT <6 months strongly correlated with the development of metastatic disease following RP [6]. Similarly, Pound et al. demonstrated that following RP, a PSADT <10 months was prognostic for the development of metastasis [4]. Furthermore, a PSADT <3 months has been shown to be prognostic for increased prostate cancer-specific mortality and decreased overall survival following RP [7,10]. Similar to the results found in RP series, a short PSADT is prognostic for poor clinical outcomes following SRT, with multiple PSADT thresholds reported to be clinically relevant. Stephenson et al. identified that a post-RP PSADT <10 months resulted in increased risk for BF following SRT [3,12]. A number of studies have looked at PSADT prior to SRT and associations with BF or metastases with thresholds selected between 3 and 12 months, although, to date, no studies have evaluated an association between PSADT and PCSM following SRT [11-18]. Interestingly, in our analysis using PSADT as a continuous variable, the strongest association as evaluated by c-index was between PSADT and PCSM (c-index 0.721) which was greater than that observed for more commonly reported end-points such as BF (0.623) and DM (0.670).

As a result, we confirm that a short PSADT post-RP is associated with poor clinical outcomes following SRT, including increased rates of BF and DM. Additionally, we establish that a rapid PSADT post-RP also increases the risk of PCSM. In our data set, analysis of hazard ratios and Kaplan-Meier curves demonstrated that a PSADT threshold of 6 months appears to be the ideal cut-point for predicting long-term response to SRT. This threshold strongly predicted BF, DM, and PCSM on multivariate analysis.

Predictive tools are needed to identify patients for whom SRT is appropriate. The Stephenson nomogram is one such tool, but only predicts for BF following SRT and has not been reported for DM, PCSM, or OM [3]. Furthermore, while the Stephenson nomogram predicts BF, the overall discriminatory power of the model leaves significant room for improvement (c-index = 0.69) [3]. In our cohort, PSADT alone had a c-index of 0.623 for predicting BF, resulting in only a minor reduction in discriminatory power compared to the Stephenson nomogram for predicting BF. This is not entirely surprising, given that PSADT is the single largest point contributor in the Stephenson nomogram, accounting for greater than 25% of the total points possible. Nevertheless, improved predictive tools are needed that can accurately and reliably predict which patients who experience BF following RP are appropriate candidates for SRT. Our data, which require validation, suggest that PSADT should be included in such nomograms, and that a threshold of 6 months would be most prognostic.

The utility of PSADT when addressing BF and PSCM after SRT is worth discussing so that SRT is not improperly selected only for patients based upon a long PSADT [23]. In our cohort, where all patients received SRT, those with a PSADT <6 months are less likely to obtain a durable response to SRT compared to those with a longer PSADT, and are most likely to die from prostate cancer. However, this does not mean that patients with a short PSADT should not receive SRT. Two large retrospective studies assessing the survival benefit of SRT for patients experiencing BF following RP [19,20], where some received SRT and others did not, identified the greatest benefit in survival from SRT in those with the shortest PSADT. Trock et al. found that comparing those treated with or without SRT, prostate cancer-specific survival was improved with SRT, but only in patients with a PSADT <6 months [20]. In contrast, Cotter et al. later demonstrated that SRT was effective in decreasing all-cause mortality both in patients with a PSADT <6 and >6 months [19]. However, when analyzing patients without co-morbidities, the risk reduction offered by SRT was approximately 1.5 fold greater for patients with a PSADT <6 months [19]. The primary outcome of interest in the study by Cotter et al. was all-cause mortality (ACM). Actuarial ACM was 49% for those receiving SRT with a PSADT <6 months, and 34% for those with a PSADT >6 months. In our cohort, median follow-up post SRT was 4 years shorter, which resulted in a lower actuarial ACM in both PSADT groups (33% for <6 months, and 20% for >6 months). However, the difference in actuarial ACM between those receiving SRT with a PSADT <6 months and >6 months was quite similar when comparing the Cotter cohort to our own (15% vs. 13%, respectively). As a result, the Trock and Cotter studies suggest that patients with a short PSADT are at greatest risk for PCSM but also most likely to benefit from SRT in terms of improved survival. Paradoxically, previous studies (11–14) and our data show that these patients are also the most likely to develop metastasis and experience a prostate cancer-specific death following SRT. These collective data are consistent with the hypothesis that following BF after RP, patients fall into two large categories: those with localized disease only and those with pre-existing metastasis. Those with a short PSADT are likely enriched for aggressive disease that could be either local or systemic. Patients with an aggressive recurrence (as represented by a short PSADT) without metastasis would have the most to gain from SRT and would be most likely to see an improvement in PCSM, whereas patients with micrometastatic spread outside of the treatment field would be least likely to benefit from SRT. In contrast, those with a long PSADT are likely to have less overall risk from their disease due to its more indolent nature and are less likely to see an improvement in PCSM with SRT (albeit more likely to have biochemical control following SRT). One can, therefore, conclude that PSADT alone is not sufficient to identify patients most likely to benefit from SRT in regard to all clinical end-points; although it does identify those at the greatest risk for PCSM who may have the most to gain if there is no evidence of metastatic disease. As a result, without improved means to identify metastasis, a short PSADT should not be used to select those not to receive SRT as this may remove this potentially curative therapy from those at greatest risk for PCSM who actually have the most to gain from SRT.

Limitations of this analysis include its retrospective design, which necessitates validation of these findings, and the fact that PSADTs could only be calculated for 48% of the SRT cohort. Unknown confounders may have been present and have the potential to bias our results if differences exist between the patients for whom PSADTs could be calculated for and those in whom we were unable to calculate PSADTs. However, no statistically significant difference existed between those with and without a determinable PSADT for any of our four primary outcomes of BF (p=0.3), DM (p=0.2), PCSM (p=0.1), and OM (p=0.1).

## Conclusions

In summary, a short PSADT after BF post-RP predicts worse outcomes after SRT. A PSADT <6 months was the best predictor of outcomes in our data set, which was particularly true for BF and DM. We also establish that a post-RP PSADT <6 months is prognostic for PCSM following SRT on both univariate and multivariate analysis. The most common prognostic nomogram for SRT uses PSADT <10 months as the cut-off point for assigning points used to predict BF following SRT. Using a PSADT of <6 months may improve the nomograms functionality. However, since these data and the Stephenson nomogram only analyzed patients treated with SRT, they can provide information regarding the likelihood of biochemical control following SRT but, without data comparing outcomes without SRT, cannot reliably predict those most likely to benefit from SRT in regard to other end-points. Indeed, those at the greatest likelihood of BF following SRT are also those most likely to progress to DM and PCSM. If SRT can prevent these events, then these patients have the potential for greatest gain from SRT. Overall, improved prognostic and predictive models are needed to identify which patients are most likely to benefit from SRT, and our findings, once validated, suggest that PSADT should be included in such nomograms with a threshold of 6 months.

## Abbreviations

ADT: Androgen deprivation therapy; ANOVA: Analysis of variance; BF: Biochemical failure; CCMI: Charlson co-morbidity index; c-index: Concordance index; DM: Distant metastasis; ECE: Extracapsular extension; HR: Hazard ratio; IQR: Interquartile range; LNI: Lymph node invasion; PCSM: Prostate cancer-specific mortality; PSA: Prostate-specific antigen; PSADT: Prostate-specific antigen doubling time; pTstage: Pathologic T stage; RP: Radical prostatectomy; RT: Radiation therapy; SM: Surgical margin; SRT: Salvage radiation therapy; SVI: Seminal vesicle involvement; OM: Overall mortality; WPRT: Whole pelvis radiation therapy.

## Competing interests

The authors declare that they have no competing interests.

## Authors’ contributions

WJ, BS, DL, CF, BF, YS, SJ, HS, DM, and FF made substantial contributions to acquisition of data. WJ, BS, DL, CF, BF, YS, MS, MS, HS, TM, GP, DH, and FF have all made substantial contributions to the conception and design of this manuscript, including analysis and interpretation of data, have been critically involved in the drafting and revising of this manuscript, and have given final approval for this final version of the manuscript to be published.
